# Atlanta Society of Medicine

**Published:** 1896-03

**Authors:** W. L. Champion

**Affiliations:** Secretary; Atlanta, Ga.


### SOCIETY REPORTS.

### ATLANTA SOCIETY OF MEDICINE.

Atlanta, Ga., February 4, 1896.

The meeting was called to order by the Vice-President, Dr. W. A. Crow.

The following members were present: Drs. Baird, Crow, Campbell, Duncan, Felder, Hurt, Kendrick, Love, Olmsted, Hutchins, Hubbard, Richardson, Spears, Stockard, Champion and F. W. McRae.

Dr. J. C. Olmsted read a paper on "Pathology of Pneumonia"; Dr. J. B. Baird on the "Treatment." (See previous pages of this issue.)

Dr. W. S. Kendrick was requested by the chairman to discuss the "Signs and Symptoms." He said as follows: "I did not expect to say anything to-night, and came to hear the papers by the gentlemen appointed to read them. In regard to the signs and symptoms of pneumonia, I would say that it is one of the easiest of all diseases to diagnose when one is acquainted with the general facts. I do not know of one that is easier when we take into consideration the symptoms of chills which usually usher in the disease, and the high temperature, etc. As has been stated, there are three divisions of the disease. The first stage is the stage in which we have an active flow of blood to the lungs, in which usually the lower lobe of the right lung is filled with blood. We have there a beginning exudation with which there is high temperature, rapid pulse, and rapid breathing. There is only one other disease which produces as profound an impression on the nervous tissues, and that is acute general peritonitis. The first stage of the pneumonia lasts from one to three days. During that stage of the engorged blood vessels we have the products of inflammation thrown out, and, as a rule, unless in an old person or a child, the lung rapidly goes into the state of red hepatization. The second stage lasts from five to seven days, in which, in typical cases, the air is entirely excluded, and this stage is accompanied by the constitutional symptoms which exist in the first stage. The pronounced constitutional symptoms last from somewhere in the middle of the second stage until the crisis, when they disappear. The last, or third, stage is resolution. Pneumonia ordinarily runs two weeks, but resolution is sometimes delayed three or four weeks. The physical signs which accompany the constitutional symptoms can be very readily marked out. In the first stage we have dullness, but not absolute dullness, as the lung is not thoroughly distended. Upon auscultation we get a harsh inspiration and a prolonged expiration. In the second stage we cannot mistake the physical signs. If you are acquainted with the normal vesicle murmur you can scarcely mistake a case of solidification in the second stage of pneumonia. Of course the second stage would show us less of distention. The two most important which make up our diagnosis are numbness and dullness. We get the dullness on account of no air being in the lung. Pleurisy with effusion is the only thing that would give dullness on percussion, but there is a way that we can distinguish between the two, and that is by auscultation. In pleurisy the vesicular murmur is cut off completely, and, of course, the bronchial tubes are rolled up and we get nothing except from the posterior root of the lung and there we get a far away murmur. We get what we call bronchial or tubular breathing. In acute pneumonia we get the tubular breathing, but not normal. In the acute pneumonia we can get the sounds at only a small space on the lower portion. We must go to the posterior portion. In the third stage, if we have attended a case, we cannot mistake it. This is the time of resolution, and it is occupied by a longer time than the second stage. It is the stage in which the constitutional symptoms have entirely passed away. Therefore, we have a more or less quiet pulse and temperature almost normal. We are then dealing simply with a lung which is returning to its normal condition. It is the stage of râles. The solid condition has to change into a liquid form before it can be absorbed. Where we have the bronchial tubes blocked up with exudation this is removed by expectoration, and consequently the expectoration is more profuse at this time. The stage of resolution is continued until completed. We get râles at the very beginning of the third stage, but as absorption goes on from day to day we get back to the stage of harsh respiration, and this lasts for some time. These are some of the many points of physical signs. The point that I would insist upon is, that to diagnose acute lobar pneumonia, if it is confined to lower right lobe, we must go to the posterior surface of the chest. My remarks have been confined entirely to the acute lobar pneumonia, as I understood that to be the form under discussion. The other forms of course give different symptoms. There would be more trouble in diagnosing the catarrhal pneumonia. Lobar pneumonia is nearly always single, but the other is double. It is simply a continuation of the inflammation of the mucous membrane on both sides of the chest. The constitutional symptoms of catarrhal pneumonia are almost entirely different. The temperature rapidly reaches its highest after the chill, and the disease is exceedingly fatal, and also we have variations of the temperature. The temperature may be up to 103 and then decline. The acute symptoms last from three to six weeks in many cases, and one lung may be more involved. It is the disease in which we do not have the rusty sputa."

DR. LOVE: "I do not feel that I can add anything to what the gentlemen have already said, except in one thing I would like to differ with Dr. Baird, and that is in the treatment of pneumonia in infants and young children. I am a strong advocate of the oil-silk jacket. I think it is most efficient, and apply it in such cases and keep it on continually. I believe that quinine in large doses will come nearer than anything else to aborting the disease."

DR. DUNCAN: "I have been very much interested in the papers and the remarks by Dr. Kendrick. My experience would cause me to differ a little with the view advanced about its being a self-limited disease. They have spoken about its lasting from ten days to weeks. I think that in many cases of the acute form it can be cut shorter than ten days. I would rather treat pneumonia than any disease I treat, but there are cases in which the right heart is overpowered by overwork. I have seen a few cases that I was sorry after they were dead that I did not bleed them. As regards the use of remedies in pneumonia, I vary in my treatment, of course, according to the patient. I treat the patient and not the pneumonia. In the last week I have seen two cases which were very different. One was a young woman with a baby three weeks old, suffering from general septic intoxication, and pneumonia set in. In this case she did not have the pneumonic cough, and did not have any cough at all, and you would not suspect pneumonia from the inflammation usually spoken of as accompanying it. Her respiration was 40 to 50 per minute, and the physical conditions on auscultation were well defined. I applied turpentine, lard, and mustard around the chest every four hours to six hours, and did not remove the previous application when renewing. I gave quinine and small doses of Dover's powder every four hours, and then quinine, salol, and sulphate of morphine to lessen the wakefulness and restlessness. The salol was used on account of excessive tympanitis. I also gave her turpentine internally. From an almost hopeless condition she has recovered slowly. In the last five days I saw another case following measles. A young lady had measles three weeks since, and after being up ten days she was attacked with pneumonia in the left lung. The attack was accompanied by severe pain, brick-dust sputa, cough. In that case, although having had fever with pulse of 100 and temperature of 102½ to 104, I gave veratrum, digitalis, quinine, camphorated Dover's powder, and capsicum. When I called the second day I could not see any improvement, except the pulse came down, but I did not know anything better to do, and so decided to continue this until the next visit. At the next visit she was very much improved, but her temperature was 103, but on the fourth day her temperature was normal and pulse 72. On the fourth day of acute pneumonia following measles I do not think better results could have been obtained with anything else than the results I obtained with veratrum. I believe we would do our patients more good if we gave veratrum in pneumonia more frequently. I have never seen a case injured by it. I approve of the course of treatment laid out by Dr. Baird. As supportive treatment I give whisky, milk punch, peptonoids, and in the latter stages I think liquid peptonoids and creosote are beneficial."

DR. HURT: "In regard to the causes and pathology mentioned by Dr. Olmsted, I must say that I fully concur with him. I think there is one point that we do not appreciate as much as we should, and that is that fifty years ago the people were not what they are to-day, and this is due to the energy and push expended in endeavoring to keep up with the latest things, and the nervous system being kept in a strain. As to the experiments made lately by the Klemperer Brothers, I think these experiments put the pneumococcus very much where the tubercular bacillus is in Koch's treatment. I think that it is in doubt, but I believe that we are on the right line if we ever succeed in getting there. I believe that, according to these gentlemen, twenty of the healthy men and women have the pneumococcus in their system, and I would like to know if they immunize one of these and then have the personi nhale strong acids, whether it will inflame the lung tissue. I confess that I am not entirely satisfied that it is dependent upon the blood condition, but I think it is somewhat due to this. As to signs, I was in hopes that Dr. Kendrick would mention something in regard to the abscess which may be present at the onset of the fever, and as the pus forms of course we have the elevated temperature, etc. As to the treatment, I think we are so widely at variance because we have not yet arrived at the definite explicit condition of the patient in pneumonia, or rather, we have not arrived at its true pathological causes. I believe that it is attendant upon a shock upon the nervous system. The history of pnemonia is that a large per cent. of pneumonic cases die, and too large to say that it is a self-evident disease; and as to its being cut short, I think it is possible to arrest pneumonic inflammation. Klemperer Brothers say that they can immunize the patient and arrest the course of the disease. If they are true about this, then I say it can be cut short. As to whether this is true, it has to be settled. From the hour the disease sets in the patient is under a strain and the blood is allowed to be filled with clots, which will be thrown upon the heart, and I think that we jeopardize the life of the patient. As regards the use of remedies, I think that at the outset it is proper to give a mercurial cathartic and some opiate with it. At the same time I would direct that flannel goods saturated with oil be applied to the chest first, and over that the thickness of two to four inches of absorbent cotton. In the first stage I would give this treatment and would keep it on, but in the third stage would remove this and make application of blister. I use as a popular remedy ammonia and small doses of anodyne, so as to make the patient comfortable and keep the blood in as healthy condition as possible. I would like to enlarge on the treatment, but I will simply say that I have bled. I will mention the case. I was called to see a farmer in March, and I would mention that he had a hernia. He was taken in the field and I reached him in four hours and found him with difficult respiration, thirty to forty-five times to the minute, great pain and high temperature. I bled him fourteen ounces and had him wrapped in cotton batting, and gave mercurial purge. The next day I found the patient able to go through a fair respiration but the pneumonia was still active. On the fourth day I removed the cotton dressing and applied blister to the whole right chest, and gave veratrum, and left instructions to give whisky later if stimulant was needed. The next visit I found him with easy respiration and he went on to rapid recovery."

DR. MCRAE: "I wish to speak very briefly on two points. One in reference to the physical signs of pneumonia. We have the rules in the early stage and in the beginning of the stage of resolution, and it seems to me that this is one sign that should not be lost sight of. As to treatment, I do not know that my views will be accepted, as they differ very materially with those that have spoken. I do not believe that the temperature has anything to do with the course of the disease or the prognosis. I do not believe in the treating of the temperature or the administration of cathartics. The giving of the antipyretic for the antipyretic effect is contrary to my idea of the treatment of the disease. I do not say that I would not give an antipyretic, but I would give perhaps in a case with anodyne. I rely on two things, and they are strychnine and alcohol, and I give them from the beginning. I believe in a mild counter-irritant. It seems to me that I have gotten marked and almost immediate effect on administration of strychnine. I rely on the administration of the strychnine, then the alcohol, and of course give nourishment. I think the strychnine has a tonic effect on the nervous system."

DR. OLMSTED: "I understood Dr. Hurt to ask if a person was immunized and then inhaled gases, what effect would it have on the lungs? I would state that this experiment has been tried, and resulted in forms of catarrhal pneumonia, but never in lobar pneumonia. I would like to say that I am sorry to hear veratrum recommended. When we remember that the organ is carrying a heavy load, to make a general practice of using a remedy, which, whatever be known about it, certainly has a depressing effect, is certainly dangerous. I admit that it may be good in some cases. We all admit that there are certain cases in which, with a full heart, the abstraction of blood is most valuable, but I think that we had better let veratrum alone."

DR. KENDRICK: "I had one point in my mind when I sat down, which I hoped some gentleman would mention and explain, and that is, what produces the crisis? Why do we have in this disease, and almost only in this disease, an abrupt termination? Why do we have a pulse dropping down from 130 to normal, temperature from 105 to normal, and a respiration of 40 to 60 dropping down to 20 or 24? These have never been explained. If we accept the theory that the cocci are the cause of the trouble, we might, by a process of reasoning, prove to our minds why it takes place. They produce themselves rapidly, propagate exceedingly fast, and if the cocci are the cause of the pneumonia, and after a certain number of days they die and pass away and the poisonous material no longer exists, we have an abrupt termination of the disease. I think that the theory of Klemperer Brothers is probably correct. In regard to treatment, I do not confirm and I do not condemn veratrum. I see no reason why it should be used. I believe with Dr. McRae, that in a number of cases of high temperature that if we do nothing for this that it will go on to recovery. I can see very readily where veratrum would be used in certain diseases, but I cannot see why it would be used after the first stage of pneumonia. In the first stage, I believe that if opium with belladonna is used in large doses, or in sufficiently safe doses, that we would do more good with it alone than with other treatment. Now, in the second stage, in which the patients usually die, there is great need to sustain a flagging heart which is overpowered. This is the reason why the disease is so fatal; they die of absolute heart failure. They have a weak heart from distended chamber in the right ventricle, and consequently this often causes them to die suddenly if they take any exercise. One case of pneumonia I had warned particularly against sitting up in bed, but contrary to my instructions he sat up soon after I left, and when I returned an hour later he had been dead half an hour. I had not given any veratrum, but simply supportive treatment with alcohol. I believe that alcohol and strychnine combined with opium are the main remedies."

DR. BAIRD: "As to phenacetin, I would say that from the standpoint of the disease I would not consider it of any consequence, but as to its effect on the patient, that is another thing. Its effect is too depressing on the patient, and, therefore, I am opposed to its use. As to veratrum, I have seen it used in a number of cases and no harm come from it, but have seen good result. If some of those who are sceptical about veratrum will give a dose and see the active heart come down to a steady beat, it will be hard to convince them that they have not done some good for the patient, although they may not have done anything for the disease. I have seen it done many a time, and some importance should be attached to it. I think it is entirely proper when used in its proper way. There is greater liability to the forming of clot in the slow heart than in the rapid heart, and therefore, of course, the veratrum should be used judiciously. The veratrum appears to strengthen the heart. I use it as long as the symptoms call for it, without regard to the stage."

DR. MCRAE reported a very interesting case of appendicitis on which he had operated in the afternoon. The patient was a young man, and he had had no previous attack, and the symptoms developed only fifty-six hours before the operation was performed. The local symptoms were tenderness and a very marked tumor. When operated found the omentum adherent to the appendix and completely surrounding it, forming a sac. The appendix was very much inflamed, and there was about a teaspoonful of pus. Death would certainly have resulted if the operation had not been performed. When operated the temperature was 101½, and pulse 120 and regular. There was no adhesion of the appendix to the wall of the intestine. Saw the patient this evening at half past six o'clock and his temperature was normal.

W. L. CHAMPION, M.D., Secretary.

---

### HOMEOPATHIC AND ECLECTIC THERAPEUTICS.

Here are some lovely illustrations of Homeopathic and Eclectic therapeutics. Dr. Frank Kraft, in the *American Homeopathist*, says that "some women in sleeping have the bad habit of placing their hands over their head. This indicates a remedy; it also sometimes denotes a tendency to falling of the womb. The remedy most prominently indicated is *puls*. *Nux vom.* is the next best remedy for the condition of putting the hands over the head in sleep; it is better for the man, *puls.* for the woman. *Ars.*, *bell.*, *platina* and *calc. carb.* are also indicated for the hands *under* the head. In asthmatic conditions or dyspnea, when the hands are placed over the head, *nux vom.* is indicated."

Slominskie, in an Eclectic journal, conveys the following useful information about prolapsus uteri:

"Alcohol burned in a cup, and patient standing over it for five minutes with limbs apart in a nightgown, will contract the uterus, and after using it for a few nights a patient suffering with prolapsus will hear the uterus flop back in its place with a noise which can be plainly heard by a bystander."

From Moore's *Common-sense Homeopathy* we learn that the "precision of homeopathic prescribing is unattainable by 'allopathy' or any other 'pathy,'" and the author arranges what he calls his "time-table of drugs," for the treatment of ague. Thus if the chill comes on from 7 to 9 o'clock *eupatorium* is to be used; if it comes from 10 to 11, *muriate of soda;* if at 11, *quinine;* if from 1 to 2, *arsenic;* if from 3 to 6, *cedron;* if at 9 P.M., *gelsemium*. What to do for chills occurring at hours not embraced by this simple table we are not informed.

